# A systematic review and meta-analysis of population-based anthrax prevalence in Africa with a one health narrative synthesis of outbreak surveillance

**DOI:** 10.3389/fpubh.2026.1792476

**Published:** 2026-04-07

**Authors:** Victor Agbajelola, Ram K. Raghavan

**Affiliations:** 1 Department of Pathobiology and Integrative Biomedical Sciences, College of Veterinary Medicine, University of Missouri, Columbia, MO, United States; 2 Department of Public Health, College of Health Sciences, University of Missouri, Columbia, MO, United States; 3 MU Institute for Data Science and Informatics, University of Missouri, Columbia, MO, United States

**Keywords:** Africa, anthrax, epidemiology, meta-analysis, one-health, surveillance

## Abstract

**Background:**

Anthrax remains a persistent public health, veterinary, and ecological challenge in Africa, sustained by fragmented surveillance systems characterized by underreporting, limited diagnostic capacity, and weak cross-sectoral coordination. The absence of integrated surveillance across human, livestock, wildlife, and environmental interfaces constrains accurate burden estimation and timely outbreak response.

**Methods:**

We conducted a systematic review and meta-analysis following PRISMA guidelines to synthesize available evidence on anthrax epidemiology in Africa. Studies published between January 2000 and February 2025 were identified from PubMed and Web of Science. Observational studies reporting primary epidemiological data in humans, livestock, wildlife, or environmental samples were eligible. Quantitative synthesis was restricted to cross-sectional studies reporting extractable prevalence data. Pooled estimates were generated using a logit-transformed random-effects model (REML), with heterogeneity assessed using *I*^2^ and τ^2^ statistics. Studies not meeting meta-analytic criteria were synthesized narratively within a One Health framework.

**Results:**

Ten cross-sectional studies comprising 19,955 samples and 2,079 confirmed anthrax cases were included in the meta-analysis. The crude aggregated prevalence was 9.88% (95% CI: 9.46%−10.30%). The pooled prevalence from the logit-transformed random-effects model was 20% (95% CI: 8%−44%). Substantial heterogeneity was observed (*I*^2^ = 98.2%), indicating marked epidemiological variability across ecological settings, host populations, and surveillance systems. Narrative synthesis further highlighted wildlife outbreaks and environmental persistence of *Bacillus anthracis*, though such studies remain comparatively scarce.

**Conclusion:**

The available evidence on anthrax in Africa is limited, geographically uneven, and highly heterogeneous. The pooled estimate should therefore be interpreted as a summary measure of reported prevalence rather than a precise continental burden estimate. These findings underscore persistent transmission within fragmented surveillance systems and support strengthened One Health–based approaches integrating human, animal, wildlife, and environmental health sectors to improve surveillance, early detection, and coordinated response across Africa.

## Introduction

Anthrax is a zoonotic disease caused by *Bacillus anthracis*, a spore-forming bacterium that persists in the environment and affects humans, domestic animals, and wildlife. In humans, the disease presents in three primary forms, cutaneous, gastrointestinal, and inhalational, each with distinct severity and mortality profiles (1). In livestock, anthrax often manifests as sudden death with hemorrhagic discharges and rapid decomposition, while in wildlife, outbreaks frequently cause mass die-offs in conservation areas (2). The remarkable resilience of B. *anthracis* spores in soil and water enables them to persist for decades, with outbreaks commonly triggered by droughts, heavy rains, and land-use changes (3, 4). These ecological and environmental drivers, compounded by anthropogenic activities, sustain continuous transmission cycles in African ecosystems. Consequently, anthrax exerts a substantial burden on public health, agriculture, and biodiversity conservation.

The disease disproportionately affects herbivorous livestock such as cattle, sheep, and goats, which serve as primary reservoirs for human infection. Transmission to humans typically occurs through direct contact with infected animals, handling contaminated animal products, or inhalation of spores (2). Livestock deaths undermine rural livelihoods, while wildlife outbreaks threaten ecotourism and conservation programs (3). Human cases are most common among farmers, butchers, veterinarians, and tannery workers, who face heightened occupational exposure risks. Case fatality rates vary widely, from about 20% in untreated cutaneous cases to nearly 100% in inhalational infections (1). Beyond the direct health consequences, anthrax outbreaks impose significant economic costs through livestock mortality, trade restrictions, and quarantine measures that disproportionately affect rural communities dependent on animal husbandry (4). Additionally, outbreaks in protected wildlife reserves, including Kruger National Park (South Africa), Etosha National Park (Namibia), and Queen Elizabeth National Park (Uganda), have caused large-scale die-offs, with severe implications for biodiversity and conservation (5–7).

Despite its endemicity, anthrax surveillance in Africa remains fragmented, underdeveloped, and inconsistently reported (4, 8). Many regions still lack reliable diagnostic capacity and coordinated data-sharing systems, and weak cross-border collaboration continues to hinder early detection and response efforts. Understandably, most published studies are country-specific, species-focused, or limited to isolated outbreak descriptions, making it difficult to discern geographically broader epidemiological patterns or identify shared risk factors (9). These gaps are further compounded by limited laboratory infrastructure, poor integration of veterinary and public health data, and the influence of environmental variables such as rainfall, temperature, and soil composition that shape anthrax persistence and distribution (10). Consequently, the true burden of anthrax across Africa remains uncertain, and control measures often remain reactive rather than preventive.

Current surveillance frameworks largely operate within disciplinary silos, viz., human health, veterinary, and environmental systems function independently rather than in coordination. This lack of integration undermines the capacity for timely outbreak detection and cross-sectoral response (11). Given the transboundary nature of anthrax transmission, a unified One Health approach is critical to bridge these gaps. The One Health concept is not new, but its practical implementation for anthrax surveillance in Africa remains limited. What is distinct in the present study is its attempt to operationalize One Health through evidence synthesis, linking human, animal, and environmental data to identify surveillance gaps and propose actionable, multisectoral strategies.

The primary aim of this study is to systematically synthesize and quantitatively summarize published evidence on anthrax occurrence in humans, livestock, wildlife, and environmental samples across Africa using a systematic review and meta-analysis of observational studies. Where data permit, pooled estimates are generated to characterize reported patterns of infection while explicitly accounting for heterogeneity and surveillance limitations. In addition, the study applies a One Health framework to interpret these findings across human, animal, and environmental interfaces, with the objective of identifying sectoral and geographic surveillance gaps and proposing integrated, One Health-driven recommendations to strengthen anthrax surveillance, early warning, and coordinated outbreak response across the continent.

## Methods

### Search strategy

This systematic review and meta-analysis followed the Preferred Reporting Items for Systematic Reviews and Meta-Analyses (PRISMA) guidelines (12) to ensure a structured and transparent synthesis of available literature on Anthrax in Africa. A comprehensive search of PubMed and Web of Science was conducted for studies published between January 1, 2000, and February 2025. The final search was performed on February 14, 2025. Eligible studies included those reporting on anthrax outbreaks, prevalence, incidence, case fatality rates, or related epidemiological outcomes in humans, livestock, wildlife, or environmental samples in Africa. The initial search strategy and preliminary organization of information were informed by consultations with two graduate students. However, all data extraction, inclusion/exclusion decisions, classification, and final analyses were performed by the listed co-authors. Any discrepancies were resolved through discussion among the co-authors, with final validation of the dataset specifically performed by the first author to ensure accuracy and consistency. Data extracted included study characteristics (authors, year, country, host species, sample size), epidemiological outcomes (prevalence, case fatality rates), and diagnostic methods used. The search strategy was designed to capture studies reporting on Anthrax outbreaks, epidemiological patterns, and surveillance approaches across Africa.

To ensure a broad yet targeted retrieval of studies, the search was constructed using a combination of subject terms and Boolean operators. The following query was applied across both databases: (“Anthrax” OR “*Bacillus anthracis*” OR “Anthrax”) AND (“Africa” OR “Sub-Saharan Africa” OR “North Africa” OR “West Africa” OR “East Africa” OR “Central Africa” OR “Southern Africa”) AND [(“outbreak” OR “epidemiology” OR “prevalence” OR “incidence”) OR (“case fatality ratio” OR CFR OR “mortality rate” OR “attack rate”)] OR (“transmission” OR “infectivity” OR “reproduction number” OR “R0”) OR (“infectious period” OR “incubation period” OR “latent period” OR “generation time”) OR (“seroprevalence” OR “serosurvey”) OR (“evolution” OR “mutation” OR “substitution”) OR (“risk factor” OR “zoonotic transmission”)) AND (“2000–2025”). These articles were then screened for relevance, and duplicates were removed before further evaluation.

### Inclusion and exclusion criteria

We included peer-reviewed studies published in English between January 1, 2000, and February 14, 2025, that reported primary epidemiological data on anthrax in humans, livestock, wildlife, or environmental samples within Africa. For quantitative meta-analysis, studies were eligible only if they: employed an observational cross-sectional design; reported population-based prevalence data; and provided extractable numerator (number of positive cases) and denominator (total sample size) information. To ensure conceptual comparability of pooled estimates, retrospective outbreak investigations, case series, and case reports were excluded from quantitative synthesis because such studies measure outbreak-period positivity rather than background population prevalence. However, these studies were retained for narrative synthesis to preserve important epidemiological context and for the generation of a distribution map.

Only studies employing recognized diagnostic approaches (serological, molecular, or bacteriological) were included. Where multiple diagnostic definitions were reported, the most stringent definition of positivity (e.g., confirmation by two assays) was extracted to enhance methodological consistency. In situations where serological data were unavailable, molecular prevalence estimates were used. Studies were excluded if they were conducted outside Africa, were not published in English, lacked primary epidemiological data, relied solely on secondary data without methodological detail, involved undefined host populations, or provided insufficient data to calculate or meaningfully interpret prevalence.

### Study selection, data extraction, and analysis

All identified studies were imported into Microsoft Excel for organization and screening. Duplicate records were removed, and the remaining unique articles were evaluated for eligibility. Two reviewers independently assessed titles and abstracts, followed by full-text screening, with disagreements resolved by consensus. The quality of each included study was appraised using the Joanna Briggs Institute (JBI) Critical Appraisal Checklist (13). Only studies that met the eligibility criteria and achieved an acceptable quality rating were retained for analysis. For each eligible study, key variables were extracted, including study characteristics (authors, year, country, study design), outbreak details (host species affected, sample size, geographic setting), and epidemiological outcomes (prevalence, incidence, case fatality rates, or attack rates). Information on surveillance strategies, such as diagnostic methods and reporting systems, was also recorded. Data was compiled in Microsoft Excel (version 2010) before analysis.

### Quantitative synthesis and statistical analysis

Crude overall prevalence was calculated by aggregating total positive cases across all eligible cross-sectional studies and dividing by the total number of samples. Exact binomial 95% confidence intervals (CI) were computed to describe the precision of this crude estimate. Meta-analysis was restricted to cross-sectional studies with extractable prevalence data. Proportions were logit-transformed prior to pooling to stabilize variances and appropriately accommodate studies with extreme values or heterogeneous sample sizes. A random-effects model using restricted maximum likelihood (REML) estimation was applied to account for between-study heterogeneity.

Heterogeneity was assessed using Cochran's Q statistic, the I^2^ statistic (representing the proportion of total variability attributable to heterogeneity rather than sampling error), and τ^2^ (between-study variance). Sensitivity analyses were conducted to evaluate robustness of pooled estimates, including the exclusion of studies with small sample sizes (*n* < 10), and the leave-one-out analyses. Influence diagnostics were assessed using externally standardized residuals, Cook's distance, and Difference in Fits (DFFITS) statistics. Potential small-study effects were explored using funnel plot visualization and Egger's regression test. Given the limited number of studies and substantial heterogeneity, these analyses were interpreted cautiously and considered exploratory rather than confirmatory. All quantitative analyses were conducted using the *metafor* and *meta* packages (14) in R (version 4.0.0).

### Narrative synthesis and one health framework

In addition to quantitative pooling, a structured narrative synthesis was conducted to incorporate studies that lacked extractable prevalence data but reported outbreak descriptions, surveillance practices, case fatality rates, ecological risk factors, or response strategies. This approach ensured that valuable contextual and operational insights were not excluded due to methodological constraints. Findings were interpreted through a One Health framework, recognizing the interconnected roles of human health, livestock systems, wildlife reservoirs, and environmental persistence in shaping anthrax transmission dynamics. This dual analytical strategy allowed pooled prevalence estimates to be contextualized within broader cross-sectoral surveillance gaps and opportunities for integrated anthrax control across Africa.

## Results

### Literature search outcome

**Figure 1:**
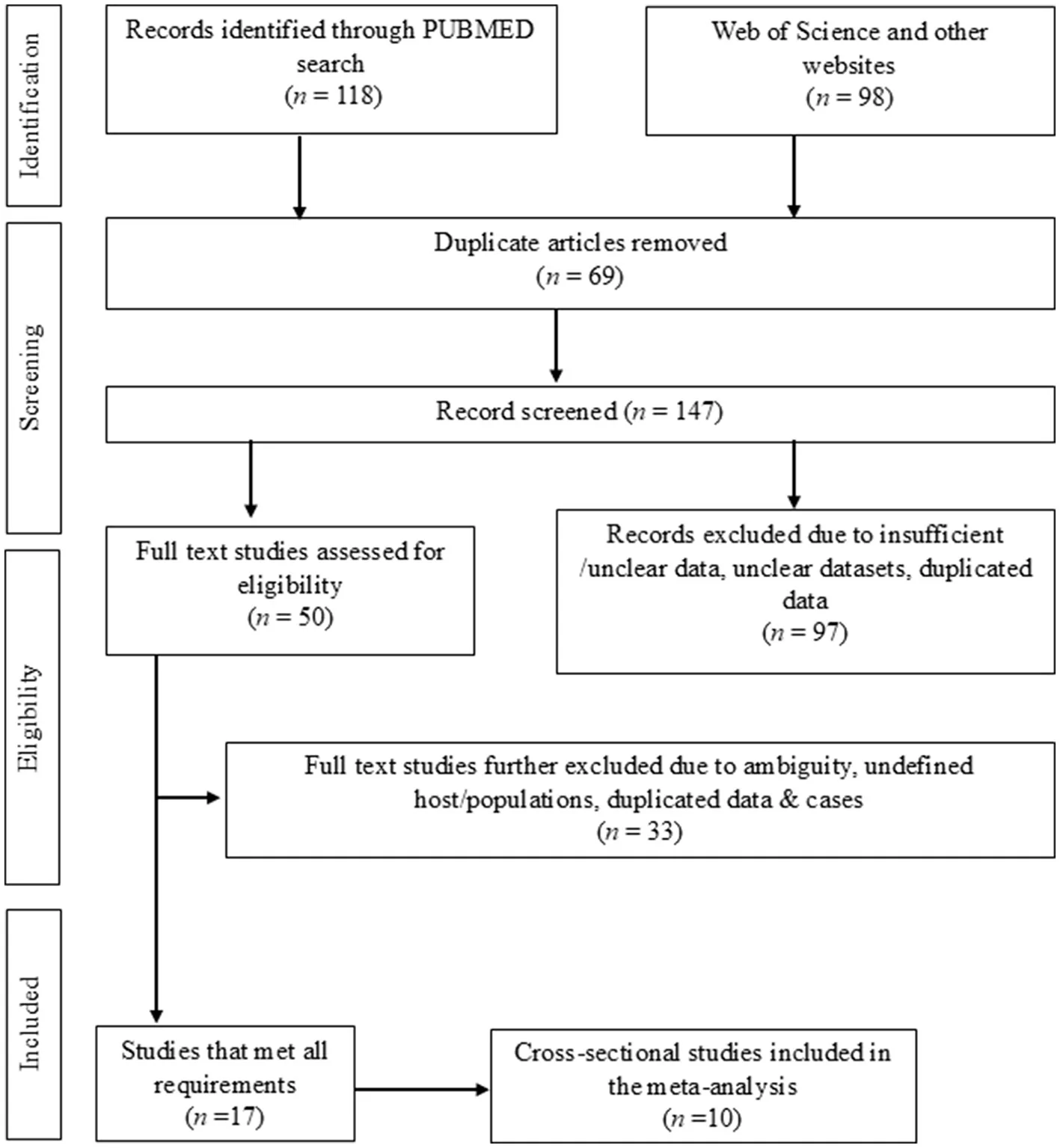
PRISMA flowchart of the article selection process.

A total of 216 records were identified from PubMed (*n* = 118), Web of Science (*n* = 80), and gray literature sources (*n* = 18). After removing 69 duplicates, 147 unique records remained for screening. Ninety-seven records were excluded due to insufficient data or aggregated datasets lacking extractable findings. Fifty full-text articles were reviewed, out of which 17 studies met the eligibility criteria; only 10 cross-sectional studies were included in the meta-analysis (Figure 1).

### Prevalence of anthrax in Africa

**Figure 2:**
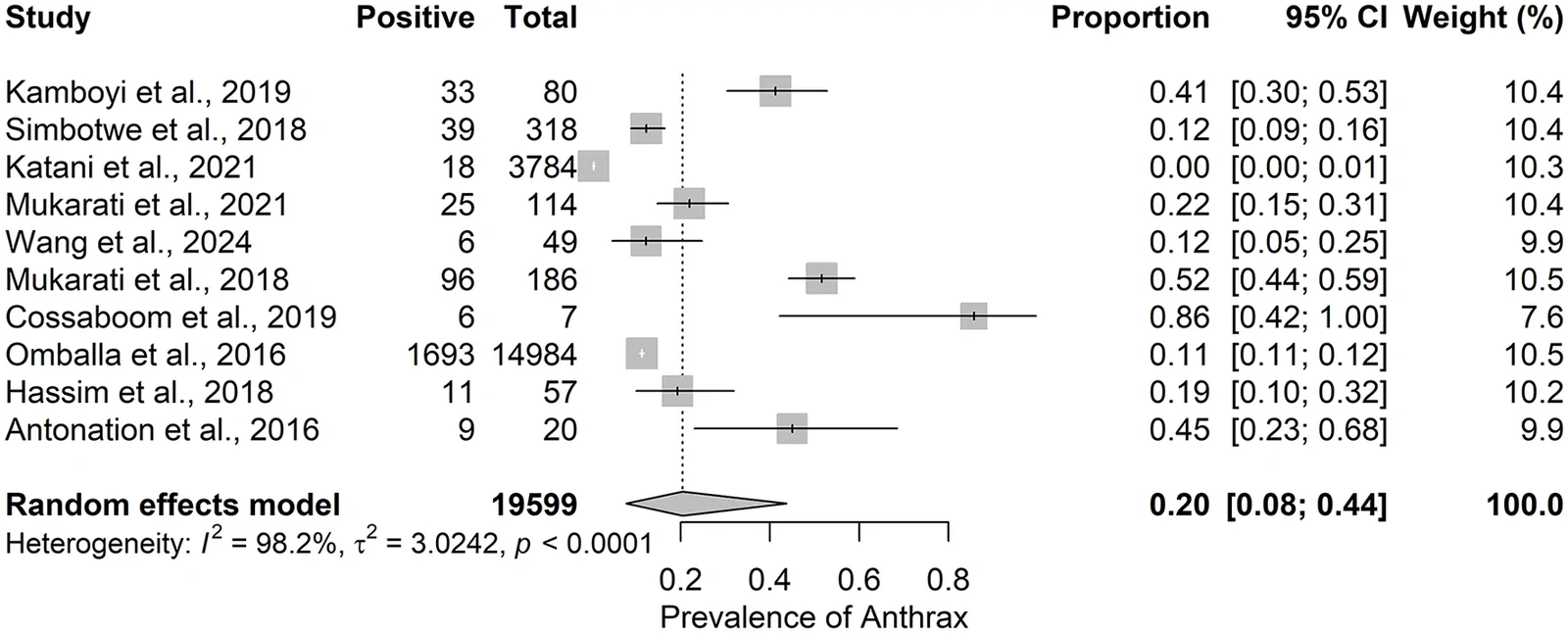
Forest plot showing the estimated pooled prevalence of anthrax in Africa.

Across the 10 studies, 19,599 samples were analyzed, yielding 1,936 positive cases, which were identified—a crude aggregated prevalence of 9.88% (95% CI: 9.46%−10.30%). Using a logit-transformed random-effects model (REML estimator), the pooled prevalence of anthrax across cross-sectional studies was 20% (95% CI: 8%−44%), with a substantial between-study heterogeneity was observed (Q = 497.92; df = 9, *p* < 0.001; *I*^2^ = 98.2%; τ^2^ = 3.02), indicating that nearly all variability between studies was attributable to true heterogeneity rather than sampling error (Figure 2; Table 1).

**Table 1:** Characteristics of prevalence studies included in the meta-analysis.

References	Country	Hosts	Positive cases	Total samples	Prevalence (%)	95% CI (%)
(15)	Zambia	Environment & biologic samples	33	80	41	30.35–52.81
(16)	Zambia	Livestock	39	318	12	8.86–16.38
(17)	Tanzania	Wildlife	18	3,784	0.47	0.28–0.75
(18)	Zimbabwe	Wildlife	25	114	22	14.72–30.64
(19)	Sierra Leone	Human	6	49	12	4.62–24.76
(20)	Zimbabwe	Dogs	96	186	52	44.20–58.98
(21)	Kenya	Wildlife	6	7	86	42.12–99.63
(22)	Kenya	Human	1,693	14,984	11	10.80–11.81
(23)	South Africa	Blowflies	11	57	19	10.04–31.91
(24)	Congo DRC	Livestock	9	20	45	23.05–68.47
*Pooled prevalence*			*2,079*	*19,599*	*20*	*0.08−0.44*

Italic values indicate the prevalence studies included in the meta-analysis.

### Sensitivity analyses

**Figure 3:**
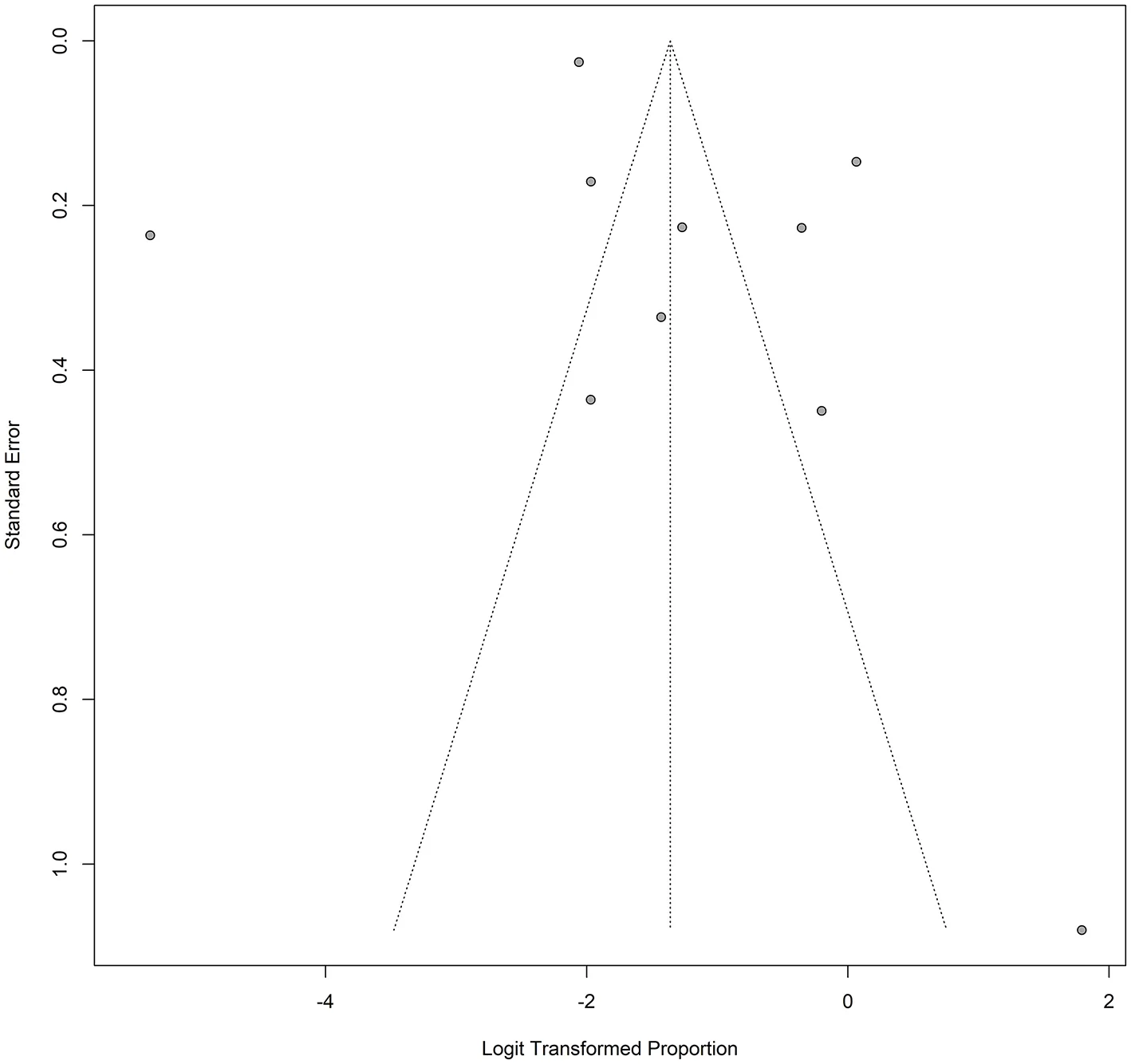
Funnel plot displaying the observed effect size of each study against the standard error of each study.

Sensitivity analyses excluding studies with small sample sizes (*n* < 10) did not materially alter the pooled estimate, and influence diagnostics did not identify any single study exerting disproportionate influence. Excluding studies with small sample sizes (*n* < 10) resulted in a pooled prevalence of 16.52% (95% CI: 6.40%−36.40%), demonstrating modest attenuation but no substantial change in interpretation. Exclusion of studies reporting 100% positivity did not materially alter the pooled estimate, which remained 20% (95% CI: 8%−44%). Influence diagnostics identified one study with elevated externally standardized residuals and Cook's distance; however, leave-one-out analysis demonstrated that removal of any single study did not materially alter the overall direction or statistical significance of the pooled estimate. When the most influential study was excluded, heterogeneity decreased (*I*^2^ = 96.76%; τ^2^ = 0.90), but the pooled estimate remained statistically significant. Egger's regression test did not indicate statistically significant funnel plot asymmetry (*z* = 1.61, *p* = 0.108). However, given the substantial heterogeneity observed, these results should be interpreted cautiously (Figure 3).

### Anthrax surveillance reports in Africa (2000–2025)

Surveillance data showed that anthrax remains endemic across multiple African regions, with outbreaks recurring over decades (Table 2). In Southern Africa, reports included livestock and human cases in Angola (2010–2018), wildlife cases in Namibia (zebras), recurrent outbreaks in Zimbabwe (2004, 2013–2014, 2018), and multi-species outbreaks in Zambia (2011–2012). Additional reports came from Malawi (human case in 2023), Mozambique (elephants and kudu in 2017), and Lesotho (livestock outbreaks, 2005–2019). In Eastern Africa, Kenya documented outbreaks in humans and wildlife (hippopotami, buffalo). Ethiopia reported thousands of human cases between 2016 and 2022. Uganda experienced major outbreaks among wildlife, including hippopotami, buffaloes, elephants, and antelope species between 2004 and 2005.

**Table 2:** Surveillance reports of anthrax cases across Africa (2000–2025).

Country	Year	Human cases (no. of deaths)	Animal cases (no. of deaths)	References
Angola	2014	NA	Livestock—150 cases (5)	(25)
Kenya	1999–2017	6	Wildlife—1,014	(26)
	2014	9	^*^Livestock	(8)
	2011–2017	NA	^*^Livestock	(27)
	2017	15 (3)	Wildlife—Hippopotamus and Buffaloes	(28)
Zimbabwe	2004	NA	Wildlife—Kudu, Nyala, Bushbuck & Antelope	(29)
	2013–2014	64	^*^Livestock—88 cases	(30)
	2012	49	NA	(30)
	2011	37	NA	(30)
	2019	NA	Wildlife—10 cases in Elephants & Buffaloes	(30)
	2018	NA	Wildlife—100 cases in Impalas	(31)
			NA	
Sierra Leone	2001–2002	NA	Wildlife—6 cases in wild chimpanzees & Gorilla	(32)
	2022	8	Livestock	(33)
Zambia	2011	NA	Domestic & Wildlife—511 cases	(28)
	2023	684 (4)	Domestic—561 cases & Wildlife—4 cases	
Cameroon	2004–2005	NA	Wildlife—Gorilla	(32, 34)
Malawi	2023	1 (1)	NA	(28)
Ethiopia	2022	9	NA	(35)
	2016–2019	1,188 (15)	NA	(36)
Morocco	2015	9	NA	(37)
Guinea	2019	5 (1)	NA	(38)
Uganda	2004–2005	NA	Wildlife—318 cases in Hippopotamuses, 60 cases in Buffalo, 1 case in African Elephant, 12 cases in Uganda Kob, 137 cases in	(39)
	2010	NA	Hippopotamus	(39)
	2022	NA (2)	Wildlife—2 cases in Waterbuck	(39)
	2023	NA (2)	NA	(28)
			NA	
Namibia	2005–2011	NA	Wildlife—80 cases in Zebra	(40)
Angola	2010–2018	26	NA	(41)
Congo DRC	2012	NA	^*^Wildlife—Gorilla & Elephant	(24)
			^*^Livestock	(24)
	2022	9 (2)	NA	(42)
Mozambique	2017	NA	^*^Wildlife—Elephants and Kudu	(43)
Lesotho	2005–2016	NA	Livestock—526 cases	(44)
	2019	50	Livestock—106 cases and 5 deaths	(45)
Ghana	2013	NA	Livestock—129 cases and 3 deaths	(46)
	2023	13 (1)	Livestock—100 deaths	(47)
Nigeria	2023	NA	Livestock—8 deaths	(48)

^*^The number of cases was not reported.

**Figure 4:**
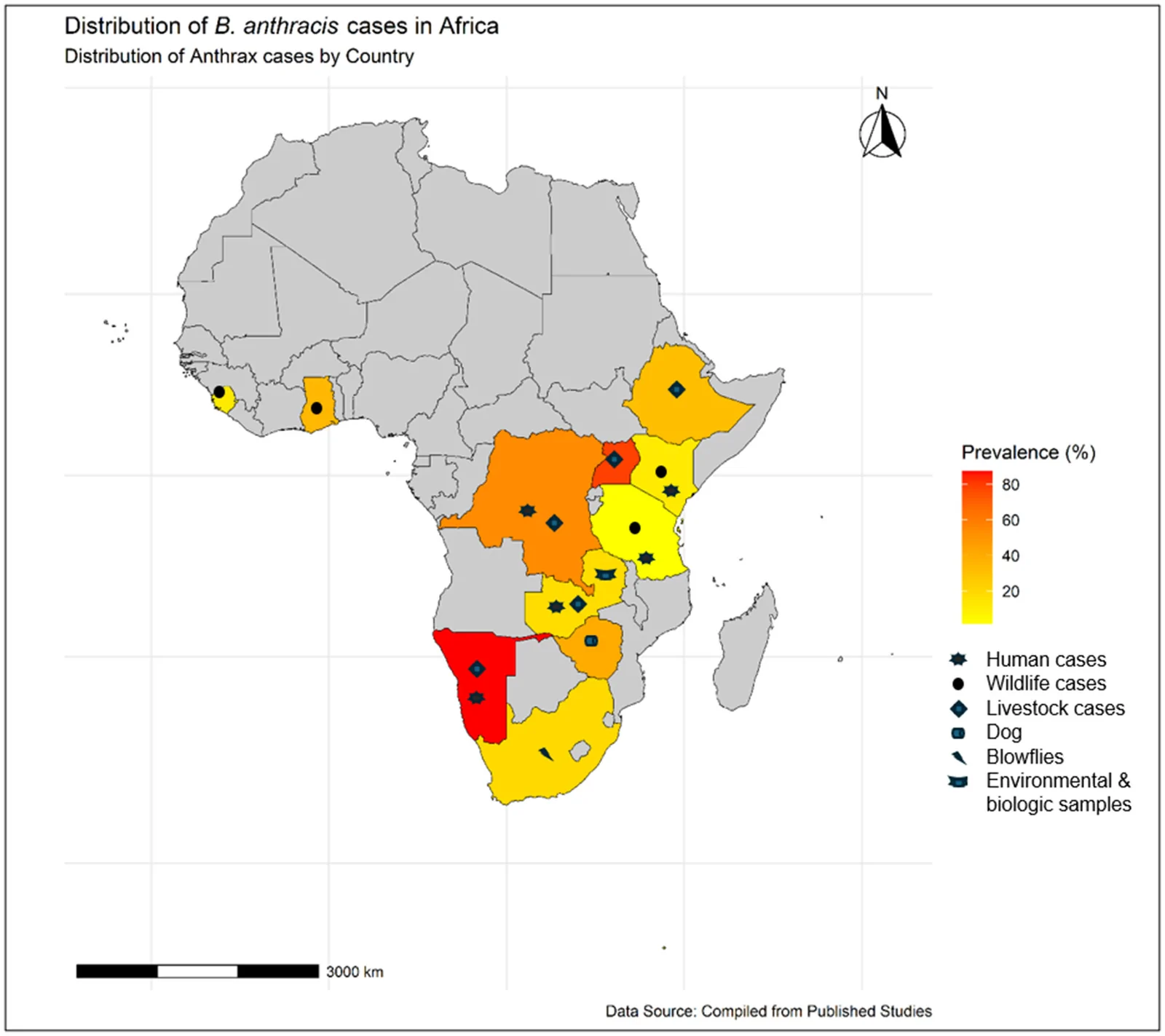
Distribution of *Bacillus anthracis* cases in Africa.

In Western Africa, Sierra Leone recorded infections in chimpanzees (2001–2002) and subsequent human and livestock cases in 2022. Cameroon documented gorilla infections (2004–2005) and additional human cases in 2019. Ghana reported livestock outbreaks in 2013 and further human and livestock cases in 2023. Nigeria also reported human and livestock cases in 2023. Morocco (North Africa) recorded human anthrax in 2015. The Democratic Republic of the Congo (DRC)—Central Africa, reported infections among gorillas, elephants, and humans, including two fatalities in 2022 (see Table 2; Figure 4).

## Discussion

This study aimed to systematically synthesize available evidence on anthrax epidemiology in Africa and to generate a pooled estimate of population-based prevalence while integrating outbreak reports within a One Health framework. By restricting quantitative synthesis to cross-sectional studies and narratively summarizing outbreak investigations, this revised analysis provides a methodologically coherent assessment of anthrax circulation across humans, livestock, wildlife, and environmental reservoirs. Overall, the findings indicate that anthrax remains an important zoonotic disease across the continent; however, the substantial heterogeneity and uneven geographic representation underscore persistent surveillance gaps rather than precise continental burden estimates.

The pooled prevalence derived from cross-sectional studies suggests notable levels of anthrax infection across host groups, though estimates varied widely by ecological and geographic context. This variability is consistent with the well-documented ecology of *Bacillus anthracis*, whose persistence in soil and environmental reservoirs facilitates sporadic but intense outbreaks. Anthrax epidemiology in Africa is known to be spatially clustered and environmentally driven, shaped by ecological conditions, livestock density, wildlife interfaces, and variability in surveillance capacity (49). Thus, the extreme heterogeneity observed *(I*^2^ = 99%) likely reflects genuine epidemiological diversity rather than a purely methodological artifact. Importantly, influence diagnostics showed that removing the most influential study reduced heterogeneity but did not materially alter the pooled estimate, supporting the robustness of the overall findings.

Although this review included studies across humans, livestock, wildlife, and environmental samples, the number of wildlife and environmental investigations was comparatively limited. This imbalance appears to reflect a genuine scarcity of structured surveillance research in these domains rather than constraints imposed by the search strategy or inclusion criteria. The search framework was intentionally broad and incorporated wildlife, environmental, and One Health–related terms; however, most identified publications focused primarily on livestock outbreaks and human cases. This pattern likely reflects surveillance priorities and resource allocation in many African settings, where livestock health and human outbreak response receive greater institutional attention than systematic wildlife or environmental monitoring. The limited representation of wildlife and environmental data therefore highlights an important surveillance and research gap that warrants targeted investment.

Livestock and human case reports identified in the narrative synthesis align with established occupational and foodborne transmission pathways and with gaps in vaccination coverage. Although causal pathways could not be directly evaluated within this meta-analysis, the persistence of cases across multiple regions highlights ongoing exposure risks. Environmental detection of *B. anthracis* spores, as reported in several included studies, further reinforces the role of ecological reservoirs in sustaining transmission cycles and the importance of environmental monitoring systems (26, 39). Similarly, wildlife outbreaks documented among chimpanzees, gorillas, and other herbivores illustrate the species- and ecosystem-specific dynamics of anthrax transmission (24, 32). These findings have implications beyond public health, extending to biodiversity conservation and communities dependent on wildlife-based economies.

Variation in prevalence estimates across diagnostic methods was explored descriptively through subgroup analyses; however, formal statistical comparisons were constrained by the small number of studies within individual diagnostic categories and the substantial heterogeneity observed. Differences in reported prevalence are more plausibly attributable to study context, host population, and outbreak status than to intrinsic differences in diagnostic performance alone. For example, bacteriological culture was frequently applied during confirmed outbreak investigations, where higher positivity rates would be expected, whereas ELISA and PCR-based methods were often used in broader surveillance settings. Accordingly, observed variability should be interpreted primarily as reflecting contextual and epidemiological differences rather than methodological superiority of one diagnostic modality.

Methodologically, this revision substantially strengthens the quantitative synthesis. Pooling was restricted to observational cross-sectional studies to ensure conceptual comparability between estimates. Outbreak investigations and case reports were excluded from pooled prevalence calculations because they measure within-outbreak positivity rather than background population prevalence. Combining such heterogeneous designs can inflate pooled estimates and artificially increase heterogeneity. Methodological guidance for prevalence meta-analysis emphasizes the importance of design comparability to ensure meaningful pooled estimates (50, 51). By restricting the analysis accordingly, the pooled prevalence more accurately reflects population-level infection rather than outbreak-period amplification.

To address statistical instability inherent in proportion meta-analysis, prevalence estimates were logit-transformed prior to pooling and analyzed using a random-effects model with REML estimation. Variance-stabilizing transformations are recommended when studies include extreme proportions or highly variable sample sizes, as they reduce bias and improve distributional assumptions (50). The logit approach provides interpretable back-transformed estimates and avoids some of the overestimation concerns associated with alternative transformations. Sensitivity analyses excluding small sample studies produced modest attenuation of the pooled estimate but did not change its overall interpretation, further supporting robustness.

Despite these methodological refinements, substantial heterogeneity persisted. Meta-regression did not identify statistically significant moderators, likely reflecting limited statistical power and unmeasured contextual factors such as surveillance intensity, diagnostic variability, outbreak reporting systems, and livestock management practices. Consequently, differences between countries should not be interpreted as definitive epidemiological contrasts but rather as reflections of uneven data availability and methodological diversity.

Assessment of small-study effects suggested possible asymmetry; however, interpretation must remain cautious given the small number of studies and extreme heterogeneity. Funnel plot methods and trim-and-fill procedures are known to perform unreliably under such conditions. Therefore, any suggestion of publication bias should be interpreted as indicative of structural reporting imbalance, particularly the overrepresentation of outbreak-associated data, rather than definitive statistical distortion. This reinforces that current literature likely captures visible outbreaks more readily than background endemic transmission.

The pooled prevalence estimate derived from cross-sectional studies should not be interpreted as representing the true continental burden of anthrax in Africa. Rather, it constitutes a statistical summary of reported prevalence across heterogeneous ecological and surveillance contexts. The substantial between-study heterogeneity observed indicates that prevalence varies widely across geographic regions, host populations, and environmental conditions. Therefore, the pooled estimate is best understood as evidence of persistent anthrax circulation and uneven surveillance capacity, rather than as a precise measure of overall burden. This distinction is critical to avoid overgeneralization of findings derived from structurally diverse study settings. Taken together, the findings of this study should not be interpreted as precise continental prevalence estimates but rather as evidence of persistent anthrax circulation occurring within fragmented and heterogeneous surveillance systems. The observed variability underscores the need for standardized diagnostic protocols, harmonized reporting structures, and integrated environmental monitoring across regions.

### One health recommendations for the surveillance of anthrax in Africa

**Figure 5:**
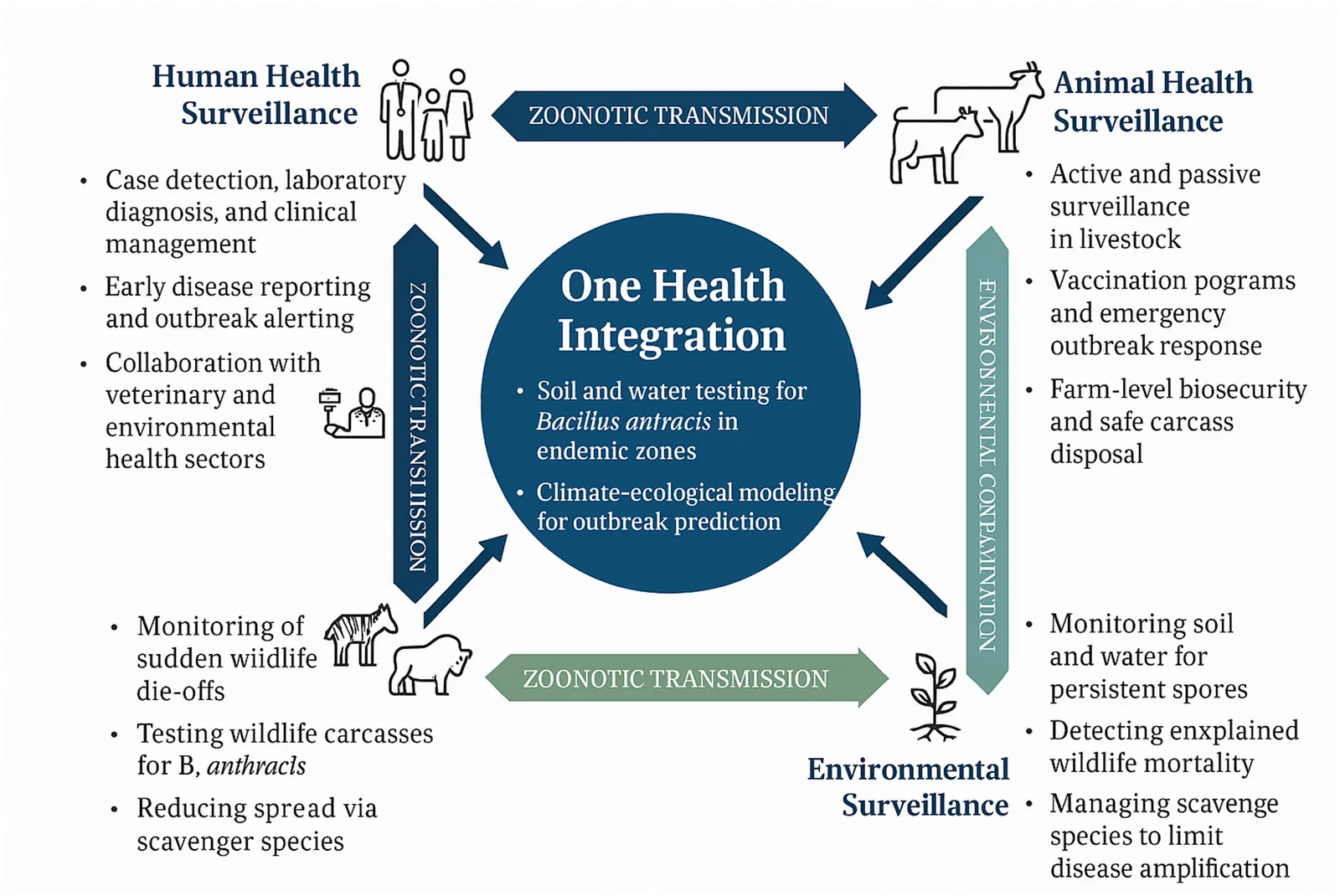
A one health surveillance framework for anthrax detection and control in Africa.

The synthesis highlights that anthrax surveillance in Africa remains fragmented across human health, veterinary, wildlife, and environmental sectors. Strengthening surveillance under a coordinated One Health framework is essential to improve early detection, outbreak response, and burden estimation. The conceptual model presented in Figure 5 outlines integrated surveillance pathways linking environmental monitoring, livestock vaccination programs, wildlife health reporting, and human case detection. By addressing structural surveillance gaps rather than focusing solely on outbreak response, African health systems can move toward more accurate epidemiological assessment and sustainable anthrax control.

This schematic illustrates the interconnections between human, animal, wildlife, and environmental health surveillance systems. Arrows represent bidirectional transmission pathways of Bacillus anthracis through zoonotic spillover and environmental contamination. The outer components detail core surveillance priorities: (i) Human health—case detection, diagnosis, early reporting, and cross-sectoral collaboration; (ii) Animal health—active and passive livestock surveillance, vaccination campaigns, and carcass disposal; (iii) Wildlife health—monitoring sudden die-offs, testing carcasses, and reducing scavenger-mediated spread; (iv) Environmental surveillance—soil and water monitoring, spore persistence detection, and controlling scavenger species. (v) At the center, the One Health Integration hub emphasizes soil and water testing, climate–ecological modeling for outbreak prediction, and cross-sectoral coordination to ensure early detection, real-time reporting, and targeted interventions.

This figure highlights how human, livestock, wildlife, and environmental surveillance can be linked through integrated data-sharing, outbreak prediction, and coordinated response strategies. This underscores the urgent need for coordinated and standardized surveillance efforts that integrate human, animal, and environmental health sectors to capture the complex transmission dynamics of anthrax and to inform effective, cross-sectoral prevention and control strategies across the continent. In the human health sector, underdiagnosis and weak reporting structures remain key barriers (49, 52); hence, integrating anthrax into existing surveillance platforms, such as the Integrated Disease Surveillance and Response (IDSR) framework, would allow earlier detection and more consistent case reporting. This should be complemented by capacity building for frontline health workers, particularly in rural areas where most cases occur. Point-of-care diagnostics, though resource-intensive, would also help reduce diagnostic delays and provide more reliable data for public health interventions. Importantly still, surveillance data from the human health sector should be routinely linked with veterinary and environmental information to provide a more complete picture of risk.

In livestock, anthrax surveillance can only be effective if it combines proactive disease monitoring with improved vaccine access, because many outbreaks are currently identified only after large-scale losses, underscoring the need for early warning systems built on regular field visits, mobile veterinary services, and the use of community animal health workers for grassroots reporting. Locally manufactured thermostable vaccines should be prioritized and subsidized to ensure affordability and accessibility. Strengthening livestock movement controls and quarantine procedures would further reduce cross-border transmission, especially in pastoralist communities where animal migration is common. Wildlife surveillance in Africa remains one of the most neglected elements, despite its central role in anthrax persistence and spillovers. Mass die-offs in protected areas highlight the need for real-time reporting systems that empower park rangers and conservation staff to detect and geo-tag suspected anthrax cases. Linking these frontline observations to national and regional databases would enable faster outbreak confirmation and response. In addition, predictive tools such as satellite-based risk mapping and soil spore monitoring can help identify ecological hotspots where surveillance and preventive measures should be concentrated (53).

Environmental surveillance should serve as the backbone that connects human, livestock, and wildlife monitoring. Systematic soil and water testing in historical outbreak zones would provide valuable data on spore persistence and guide targeted decontamination strategies. At the community level, education programs on carcass disposal, safe meat handling, and grazing management can reduce both human infections and environmental contamination, creating feedback loop between local practices and formal surveillance systems. Overall, strengthening anthrax surveillance in Africa requires more than incremental improvement. It calls for deliberate integration of existing health, veterinary, and environmental systems under a unified One Health strategy. By connecting data streams, standardizing diagnostic practices, and investing in cross-sectoral early warning networks, African countries can build a surveillance architecture that is both responsive and sustainable. Such a system would not only improve anthrax detection and control but also provide a model for addressing other neglected zoonoses at the human–animal–environment interface.

To enhance feasibility in low-resource African settings, proposed One Health strategies can be conceptualized along a continuum of short-term and long-term actions. Immediately implementable measures include strengthening passive surveillance reporting, improving coordination between veterinary and public health sectors, enhancing community risk communication, and conducting targeted livestock vaccination in high-risk areas. These interventions can often be integrated within existing infrastructure. In contrast, longer-term structural strategies—such as development of harmonized cross-sectoral databases, expansion of environmental monitoring capacity, sustained wildlife health surveillance systems, laboratory standardization, and formal institutionalization of One Health governance frameworks—require sustained investment and policy-level commitment. Distinguishing between pragmatic short-term steps and broader structural reforms ensures that recommendations remain context-sensitive and operationally realistic.

### Study limitations

The available evidence base remains geographically uneven, with cross-sectional prevalence studies identified from only a subset of African countries, leaving large regions underrepresented. As a result, pooled estimates may reflect variations in surveillance capacity rather than true epidemiological distribution. Although quantitative synthesis was restricted to cross-sectional studies to enhance comparability, substantial heterogeneity persisted (*I*^2^ = 99%). Differences in ecological settings, host species, sampling strategies, diagnostic methods, and outbreak contexts likely reflect genuine epidemiological variability across Africa but limit the interpretability of a single summary estimate.

In addition, assessment of small-study effects using funnel plots and Egger's regression was constrained by the limited number of studies and extreme heterogeneity, reducing the reliability of formal publication bias evaluation. The literature may over represent severe or outbreak-associated events, while underreporting driven by limited diagnostic infrastructure and fragmented surveillance systems remains possible. Accordingly, the pooled prevalence should not be interpreted as a precise continental burden estimate, but rather as a synthesis of available data indicating persistent transmission within structurally uneven surveillance systems and underscoring the need for more systematic, standardized, and representative data collection across Africa.

## Conclusion

This review shows that anthrax persists as a zoonotic disease in Africa but is unevenly studied, with wide variability across hosts, regions, and diagnostic approaches. The fragmented evidence base requires cautious interpretation of pooled findings and highlights major gaps in surveillance. Strengthening monitoring through early detection, standardized diagnostics, and improved reporting, within a One Health framework that links human, animal, and environmental health, is essential. Expanded surveillance in underrepresented regions and further research will be critical to refining understanding and guiding more effective, context-specific control strategies.
