# Correction: A systematic review and meta-analysis of population-based anthrax prevalence in Africa with a one health narrative synthesis of outbreak surveillance

**DOI:** 10.3389/fpubh.2026.1979617

**Published:** 2026-09-09

**Authors:** Victor Agbajelola, Ram K. Raghavan

**Affiliations:** 1 Department of Pathobiology and Integrative Biomedical Sciences, College of Veterinary Medicine, University of Missouri, Columbia, MO, United States; 2 Department of Public Health, College of Health Sciences, University of Missouri, Columbia, MO, United States; 3 MU Institute for Data Science and Informatics, University of Missouri, Columbia, MO, United States

**Keywords:** Africa, anthrax, epidemiology, meta-analysis, one-health, surveillance

In the published article, the funding statement was incorrectly given as:

The author(s) declared that financial support was received for this work and/or its publication. This research was sponsored by the Department of the Army, U.S. Army Contracting Command, Aberdeen Proving Ground, Natick Contracting Division, Ft Detrick MD (Award # W911S expressed R2210004).

The correct funding statement is:

The author(s) declared that financial support was received for this work and/or its publication. This research was sponsored by the Department of the Army, U.S. Army Contracting Command, Aberdeen Proving Ground, Natick Contracting Division, Ft Detrick MD (Award # W911SR2210004). Any opinions, findings, and conclusions or recommendations expressed in these materials are those of the author(s) and do not necessarily reflect the position or policy of the Government, and no official endorsement should be inferred.

The original version of this article has been updated.

